# Direct detection of extended-spectrum beta-lactamases (CTX-M) from blood cultures by LC-MS/MS bottom-up proteomics

**DOI:** 10.1007/s10096-017-2975-y

**Published:** 2017-04-10

**Authors:** F. Fleurbaaij, W. Goessens, H. C. van Leeuwen, M. E. M. Kraakman, S. T. Bernards, P. J. Hensbergen, E. J. Kuijper

**Affiliations:** 1 Department of Medical Microbiology, Leiden University Medical Center, PO Box 9600, 2300 RC Leiden, The Netherlands; 2 Department of Medical Microbiology and Infectious Diseases, Erasmus University Medical Center, 3015 CN Rotterdam, The Netherlands; 3 Center for Proteomics and Metabolomics, Leiden University Medical Center, 2333 ZA Leiden, The Netherlands

**Keywords:** Proteomics, ESBL, Beta-lactamase, Blood cultures, Mass spectrometry

## Abstract

Rapid bacterial species identification and antibiotic susceptibility testing in positive blood cultures have an important impact on the antibiotic treatment for patients. To identify extended-spectrum beta-lactamases (ESBL) directly in positive blood culture bottles, we developed a workflow of saponin extraction followed by a bottom-up proteomics approach using liquid chromatography coupled to tandem mass spectrometry (LC-MS/MS). The workflow was applied to positive blood cultures with *Escherichia coli* and *Klebsiella pneumoniae* collected prospectively in two academic hospitals over a 4-month period. Of 170 positive blood cultures, 22 (12.9%) contained ESBL-positive isolates based on standard susceptibility testing. Proteomic analysis identified CTX-M ESBLs in 95% of these isolates directly in positive blood cultures, whereas no false positives were found in the non-ESBL producing positive blood cultures. The results were confirmed by molecular characterisation of beta-lactamase genes. Based on this proof-of-concept study, we conclude that LC-MS/MS-based protein analysis can directly identify extended-spectrum beta lactamases in *E. coli* and *K. pneumoniae* positive blood cultures, and could be further developed for application in routine diagnostics.

## Introduction

Infections caused by antibiotic resistant Gram-negative bacteria are an increasing problem worldwide. In the Netherlands, resistance towards third generation cephalosporins through extended-spectrum beta-lactamases (ESBLs) is the most frequently found antibiotic resistance of medical importance [1]. Unrecognised, infections with ESBL-producing bacteria pose a serious threat, as they are associated with high morbidity and mortality rates [2]. *Escherichia coli* and *Klebsiella pneumoniae* are reported among the main representatives of ESBL-producing bacteria. In the Netherlands, ESBL prevalence is approximately 10% among infected patients [3].

Extended-spectrum beta-lactamases are a group of beta-lactamases which can also hydrolyze third generation cephalosporins. The detection of these ESBL-enzymes is currently provided indirectly by the results of standard susceptibility testing of cultured bacteria, followed by a phenotypic confirmation assay or a genetic test. Direct detection of the enzyme responsible would provide molecular information regarding the phenotype. Protein analysis by way of mass spectrometry has changed microbiological practice in recent years through the introduction of Matrix-Assisted Laser Desorption Ionisation-Time of Flight Mass Spectrometry (MALDI-TOF MS) for species identification [4]. However, the inherent limitations of these instruments such as the limited dynamic range and resolution limit the general applicability to accurately detect the presence of ESBL, particularly in identifying the nature of the underlying enzyme.

Peptide analysis by bottom-up proteomics is commonly used to directly identify proteins and can be used for in-depth proteomic characterisation of resistant bacteria, often using multi-dimensional protein and/or peptide fractionation techniques [5–7]. However, straight analysis of proteolytic digests of total cellular protein extracts also allows to directly identify resistance-related proteins such as beta-lactamases [8, 9]. This leads to shorter analysis times compared to comprehensive proteome studies while maintaining the inherent specificity of directly identifying the protein of interest. Previously we developed such a proteomic platform for the direct detection of OXA-48 and KPC carbapenemases in bacterial cultures of clinical isolates [10–12].

A significant reduction in analysis time would be achieved when bacterial beta-lactamases could be directly analyzed in positive blood cultures. Therefore, the aim of this study was to develop a LC-MS/MS based bottom-up proteomics workflow to identify ESBL-producing *E. coli* and *K. pneumoniae* directly in blood cultures and to test the performance of this workflow in a proof-of-principle study using clinical blood culture samples collected in a prospective study.

## Materials and methods

### Design of study

This study was designed to evaluate the use of proteomic analysis by LC-MS/MS for the detection of extended-spectrum beta lactamases directly from positive blood culture bottles that grow *E. coli* or *K. pneumonia*, during a prospective study. Two academic centers participated in the study: the Erasmus University Medical Center in Rotterdam and the Leiden University Medical Center in Leiden. During a period of 4 months (July–October 2015), all positive blood cultures with *K. pneumoniae* or *E. coli*, were included in the study.

### Comparison of sample preparation methods

Two methods were evaluated for the analysis of bacterial proteins in blood cultures, serum separator tubes and a differential lysis protocol using saponin. For this purpose, negative blood culture bottles were spiked with different amounts of liquid broth culture of *E. coli* BL21(DE3) pLysS to mimic different bacterial densities in positive blood cultures.

Serum separator tubes feature a gel through which red blood cells can migrate while bacteria are pelleted on top of the gel. Four mL from a spiked blood culture bottle were applied to the tubes (Becton Dickinson, Breda, The Netherlands) and the mixture was centrifuged at 6000 g for 10 min. Serum was removed and the pellet was washed twice with 1 mL of phosphate buffered saline (PBS) followed by a 5 min centrifugation at 6000 g. The bacterial pellet on top of the gel was resuspended in 100 μL PBS and transferred to an Eppendorf tube. The gel was rinsed with 100 μL PBS another three times to recover any residual bacteria and this was added to the vial. The resulting bacterial suspension was centrifuged at 10,000 g for 1 min and the supernatant was removed. 100 μL of 50% trifluoroethanol (TFE) solution was added for protein extraction and solubilisation. This suspension was sonicated in an ultrasound water bath for 2 min. Suspensions were heated to 60 °C for an hour. The resulting lysates were then subjected to protein digestion.

For the saponin protocol, 4 mL of the spiked blood culture bottle was mixed with 1 mL of a saponin (Sigma-Aldrich, Zwijndrecht, The Netherlands) stock solution (5% *w*/*v*, final concentration 1% *w*/*v*). The mixture was vortexed, incubated at room temperature for 5 min and centrifuged at 6000 g for 10 min. The cell pellet was washed three times with 1 mL PBS and centrifuged at 10,000 g for 1 min and, following the final centrifugation step, re-suspended in 100 μL 50% TFE solution. The suspension was sonicated using an ultrasound water bath for 2 min. Suspensions were heated at 60 °C for an hour. The resulting lysates were stored at −80 °C until further analysis.

### Blood culture and species identification

Blood cultures were drawn as part of normal clinical routine. A sample of 8–10 mL of blood was used per bottle (Bactec Plus Aerobic and Bactec Plus Anaerobic, Becton Dickinson, Breda, The Netherlands) for blood culturing (Bactec FX, Becton Dickinson, Breda, The Netherlands). Bacterial species in positive cultures were identified directly from 1 mL blood culture by MALDI-TOF MS analysis (Microflex, Bruker Daltonics, Bremen, Germany) according to an in-house developed protocol adapted from literature [13]. All positive flagged blood cultures were stored at 4 °C and processed within 48 h using the saponin protocol described above.

### Susceptibility testing

Susceptibility testing was performed with VITEK 2 (bioMérieux, Marcy l’Etoile, France). The presence of ESBLs was performed using the combination disk diffusion tests (Rosco Diagnostica A/S, Taastrup, Denmark) according to the Dutch guideline [14]. Disks used were ceftazidime (30 μg), ceftazidime + clavulanate (30 + 10 μg), cefotaxime (30 μg) and cefotaxime + clavulanate (30 + 10 μg). ESBL production was considered positive if the zone diameters around one or both of the combination disks was ≥5 mm compared to the corresponding antibiotic-only disk. E-tests for ceftazidime, cefotaxime and meropenem were performed on Mueller Hinton E agar media according to the recommendations of EUCAST (http://www.eucast.org/clinical_breakpoints/).

### In-solution protein digestion

Stored lysates (at −80 °C) were thawed for further processing for bottom-up proteomics. Reduction was performed with dithiothreitol (DTT, final concentration 2.5 mM in 25 mM ammonium bicarbonate) at 60 °C for 15 min. Alkylation was performed in the dark with iodoacetamide (final concentration 5.5 mM in 25 mM ammonium bicarbonate) for 15 min. Following alkylation the samples were digested overnight using sequencing grade modified trypsin (12.5 ng/μl, Promega, Leiden, The Netherlands). The next day the resulting digests were lyophilized and reconstituted in 0.5% trifluoroacetic acid (TFA) for pre-column trapping during LC-MS/MS analysis.

### Molecular characterisation

All ESBL positive isolates (*n* = 22) were analyzed for the presence of beta-lactamase genes. An in-house real-time multiplex *bla*_CTX-M_ PCR was used for analysis of the specific CTX-M groups. For primer design, an alignment of the available *bla*_SHV_ gene sequences from GenBank® was made using the AlignX program (Vector NTI Advance 11, Invitrogen). Primers and probes were developed in-house using Beacon Designer (Premier Biosoft, Palo Alto, U.S.A.). Subsequently, beta-lactamase gene *bla*_SHV_ was amplified using PCR and further investigated by nucleotide sequence analysis [15]. All primers and probes used in this study are listed in Table 1. These molecular assays have been developed and internally validated at the LUMC and are used in daily routine.

**Table 1:** Primers and probes used in this study

Target	Forward primer	Reverse primer	Probe (5′ to 3′)
	(5′ to 3′)	(5′ to 3′)	
*bla*_SHV_	GCCGGTTATTCTTATTTGTCGC	ATGCCGCCGCCAGTCA	
*bla*_CTX-M1 family_	CTGACYTKGTTAACTATAATC	GTGAGMAATCAGCTTATTC	CCACGTTATCGCTGTACTGTAG
*bla*_CTX-M2 family_	ACCTGGTTAACTACAATC	GCAGTATTGTCGCTATAC	ATTGCGGAGAAACACGTTAACG
*bla*_CTX-M9 family_	CCGATCTGGTTAACTACA	GGCAATCAATTTGTTCATG	AACACGTCAACGGCACAATG
*bla*_CTX-M26 family_	CTCAGACTTGRTTAACTACA	GCAGTATTATCGCTGTAC	CGTCAATGGCACGATGACAT

### LC-MS/MS analysis and data processing

Peptide mixtures were analyzed using nano reversed-phase liquid-chromatography coupled to tandem mass spectrometry (nano LC-MS/MS). The nano-LC system (Ultimate 3000 RSLCnano, Dionex) combines a 2-cm Acclaim PepMap 100 guard column with an Acclaim PepMap RSLC column (C18, 75 μm × 50 cm with 2 μm particles). A multi-step gradient going from 5 to 55% B in 180 min was used (solvent A being 0.1% formic acid in water and solvent B 0.1% formic acid in 80% acetonitrile) at a rate of 300 nl min^−1^. Mass spectrometry analysis was carried out on a maXis Impact UHR-TOF-MS (Bruker Daltonics) in data dependent MS/MS mode, with precursors ranging from *m/z* 300–1200. After MS/MS analysis precursors were excluded from selection dynamically for one minute.

Raw data were converted to Mascot Generic Files (MGF) and analyzed by database searching using the Mascot algorithm (Mascot 2.5.1, Matrix Science, London, UK) using Mascot Daemon 2.5.1. To ensure a comprehensive search of all beta-lactamases, a custom database was prepared. This database consists of *in-silico* translated reference genomes for *K. pneumoniae* (http://www.ncbi.nlm.nih.gov/genome/815?genome_assembly_id=168877) and *E. coli* (http://www.ncbi.nlm.nih.gov/genome/167?genome_assembly_id=161521), supplemented with a comprehensive list of beta-lactamases continued from the former Lahey database (December 2015, ftp://ftp.ncbi.nlm.nih.gov/pathogen/betalactamases/Allele-prot.fa). Searches were carried out with the following parameters: precursor mass tolerance was 0.05 Da and MS/MS tolerance 0.8 Da. Carbamidomethylcysteine was set as a fixed modification, with methionine oxidation as variable modification. Trypsin was designated as an enzyme with a maximum allowed number of missed cleavages of two. The False Discovery Rate (FDR) was set at 0.01 at the peptide level based on decoy database searches.

## Results

### Optimization of sample extraction

Two different sample preparation protocols were compared with respect to the overall number of protein identifications and the ease of use of the method. For this purpose, we used negative blood culture bottles spiked with different amounts of liquid broth culture of *E. coli* BL21(DE3) pLysS cells to mimic different bacterial densities in positive blood cultures. The number of successful protein identifications was determined at 3.0 × 10^7^ and 3.0 × 10^8^ CFU using a sample preparation by serum separator tubes and by differential lysis protocol using saponin. As a reference, the protocols were also applied to the bacterial suspensions used for inoculation of the blood cultures, using 1.0 × 10^7^ CFU. Results of all analyses were searched independently against the bacterial database and, for human proteins, against the human database. Table 2 summarises the results for the proteomic comparisons of both protocols. The sample containing 3.0 × 10^8^ CFU performed better in the saponin protocol. Since this procedure is less laborious we treated all subsequent positive blood cultures by the saponin protocol. A number of variables of the saponin lysis protocol were tested including centrifugation speed and duration, saponin concentration and number of washing steps. No significant improvements were made and the protocol therefore remained unchanged.

**Table 2:** Comparison of the number of protein identifications using two different sample preparation protocols for LC-MS/MS analysis of bacterial protein extracts from blood cultures

Sample	Bacterial proteins	Human proteins
Reference	566	16
Saponin 3.0 10^7^ CFU	196	165
Saponin 3.0 10^8^ CFU	477	135
SST 3.0 10^7^ CFU	199	145
SST 3.0 10^8^ CFU	288	82

Samples were spiked with 3.0 10^7^ or 3.0 10^8^ CFU obtained from liquid broth culture. Saponin: differential lysis protocol. SST: Serum separator tube protocol. As a reference, a suspension containing 1.0 10^7^ CFU was prepared from the same liquid culture that was used to spike the negative blood cultures

### Blood culture collection and susceptibility testing

During a period of four months, positive blood cultures with *E. coli* or *K. pneumoniae* were collected prospectively (Table 3). In total, 170 positive blood cultures were collected. Of these, 125 (73.5%) contained *E. coli* and 45 (26.5%) *K. pneumoniae.* Following susceptibility testing of cultured isolates, 22 isolates (12.9%, 18 *E. coli* and 4 *K. pneumoniae*) were confirmed as ESBL-positive with the combination disk diffusion test.

**Table 3:** Collection of positive blood cultures

Origin	Positive blood cultures					
	*E. coli*	no. ESBL+ (%)	*K. pneu.*	no. ESBL+ (%)	Total	no. ESBL+ (%)
ErasmusMC	57	8 (14.0)	22	3 (11.1)	79	11 (13.9)
LUMC	68	10 (14.7)	23	1 (4.3)	91	11 (12.1)
Sum	125	18 (14.4)	45	4 (8.9)	170	22 (12.9)

Samples were collected in two university medical centers in the Netherlands: the Erasmus MC in Rotterdam and the Leiden University Medical Center (LUMC) in Leiden. Presence of ESBLs was determined using phenotypical susceptibility testing

### Results from bottom-up proteomics analysis

**Fig. 1:**
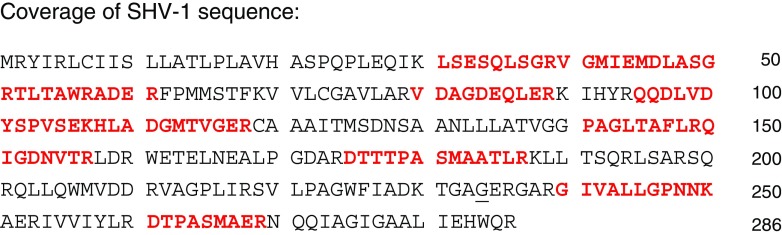
Coverage of SHV-1 sequence. Identified peptides by LC-MS/MS analysis are highlighted when they matched to the sequence of the SHV-1 beta-lactamase. The glycine at Ambler position 238 (*underlined*) is specific for the SHV-1 sequence, while SHV-2 type extended-spectrum beta-lactamases have a serine in this position. This peptide was not observed in LC-MS/MS analysis making it not possible to distinguish between the beta-lactamase types

All 22 blood cultures with ESBL positive isolates were selected for proteomic analysis, as well as 44 randomly selected ESBL negative blood cultures. Preparation of the 66 blood cultures for LC-MS/MS analysis was performed blind with regards to the results of the phenotypic testing. Following LC-MS/MS analysis, the resulting spectra were searched against the in-house generated database (see materials and methods) featuring a comprehensive list of beta-lactamases as well as *K. pneumoniae* and *E. coli* proteomes. In a typical analysis of one positive blood culture bottle, 400–800 bacterial proteins were identified. Table 4 summarises the results for the phenotypically ESBL positive blood cultures (*n* = 22). In all results obtained by MS, the detected β-lactamase was always in the top 10% of the total number of identified bacterial proteins in a sample, sorted by identification score. In 21 out of 22 of the ESBL positive isolates a cefotaximase (CTX-M) was identified. Protein sequence coverage based on identified peptides varied from 38% to 88%. This coverage allows for the mapping of the identified cefotaximases into one of six established lineages [16], named after their archetypical enzymes. In our collection, only members of groups CTX-M-1 and CTX-M-9 were found. In one *K. pneumoniae* isolate (Table 4, number 15) no cefotaximase was found. A SHV type beta-lactamase was identified with 33% coverage (Fig. 1). Like with the cefotaximases, this protein was a top 10% identification among all bacterial proteins identified. From 148 ESBL-negative *K. pneumonia* or *E. coli* positive blood cultures, 44 were randomly selected and also analyzed by LC-MS/MS. In none of these samples, extended-spectrum beta-lactamases were found.

**Table 4:** Resistance profile and LC-MS/MS-based ESBL identification in ESBL positive blood cultures

Culture	Origin	Species	Resistance profile			β-lactamase detection	
			MIC (mg/L)			LC-MS/MS	PCR
			CTX	CZD	Mero		
1	LUMC	*E. coli*	>32	8	0.023	Group 1 CTX-M	Gr 1 CTX-M
2	LUMC	*E. coli*	>32	24	0.032	Group 1 CTX-M	Gr 1 CTX-M
3	LUMC	*E. coli*	>32	3	0.023	Group 9 CTX-M	Gr 9 CTX-M
4	LUMC	*E. coli*	>32	3	0.023	Group 9 CTX-M	Gr 9 CTX-M
5	LUMC	*E. coli*	>32	8	0.012	Group 1 CTX-M	Gr 1 CTX-M
6	LUMC	*K. pneumoniae*	>32	32	0.032	Group 1 CTX-M	Gr 1 CTX-M; SHV-1
7	LUMC	*E. coli*	>32	1.5	0.023	Group 1 CTX-M	Gr 1 CTX-M
8	LUMC	*E. coli*	>32	4	0.023	Group 1 CTX-M	Gr 1 CTX-M
9	LUMC	*E. coli*	>32	6	0.023	Group 1 CTX-M	Gr 1 CTX-M
10	LUMC	*E. coli*	>32	2	0.023	Group 1 CTX-M	Gr 1 CTX-M
11	LUMC	*E. coli*	>32	6	0.023	Group 1 CTX-M	Gr 1 CTX-M
12	Erasmus MC	*E. coli*	>32	32	0.023	Group 1 CTX-M	Gr 1 CTX-M
13	Erasmus MC	*E. coli*	8	0.25	0.023	Group 9 CTX-M	Gr 9 CTX-M
14	Erasmus MC	*E. coli*	>32	6	0.023	Group 1 CTX-M	Gr 1 CTX-M
15	Erasmus MC	*K. pneumoniae*	8	32	0.032	SHV^a^	SHV-12
16	Erasmus MC	*E. coli*	>32	0.75	0.012	Group 9 CTX-M	Gr 9 CTX-M
17	Erasmus MC	*K. pneumoniae*	>32	8	0.032	Group 1 CTX-M	Gr 1 CTX-M; SHV-11
18	Erasmus MC	*E. coli*	>32	12	0.032	Group 1 CTX-M	Gr 1 CTX-M
19	Erasmus MC	*K. pneumoniae*	>32	48	0.094	Group 1 CTX-M	Gr 1 CTX-M; SHV-1
20	Erasmus MC	*E. coli*	>32	8	0.023	Group 1 CTX-M	Gr 1 CTX-M
21	Erasmus MC	*E. coli*	>32	8	0.023	Group 1 CTX-M	Gr 1 CTX-M
22	Erasmus MC	*E. coli*	>32	48	0.023	Group 1 CTX-M	Gr 1 CTX-M

*CTX* cefotaxime, *CZD* ceftazidime, *Mero* meropenem

MIC (mg/L) values were determined using E-tests.^a^The MS/MS data was inconclusive about the positive identification of this SHV as an ESBL because the single peptide necessary to discriminate between an ESBL and non-ESBL was not identified. The sequencing of the PCR product confirmed that this was an ESBL. See text for further explanation

### Molecular characterisation of ESBL positive isolates

To confirm the identity of the ESBLs identified with LC-MS/MS based proteomics, molecular characterisation of all phenotypically ESBL positive isolates was performed (Table 4). All CTX-M identifications were verified with PCR. In isolate 15, the LC-MS/MS identified a SHV-enzyme which was confirmed by PCR as an ESBL, namely, SHV-12. Three non-ESBL SHV beta-lactamases were identified by PCR in the *K. pneumoniae* isolates.

## Discussion

In this study we developed a novel proteomic workflow for the direct identification of ESBLs in positive blood culture bottles. To evaluate the performance of our approach, a proof-of-principle prospective study was performed in two academic hospitals. In 22 positive blood cultures with phenotypically ESBL producing *E. coli* or *K. pneumoniae*, we identified 21 isolates containing a CTX-M and one isolate containing a SHV beta-lactamase, although the latter could not unambiguously be identified because the single peptide necessary to discriminate between an ESBL and non-ESBL was not identified. In the set of positive blood cultures with ESBL-negative *E. coli* or *K. pneumoniae*, no ESBLs were identified by LC-MS/MS analysis. This demonstrates a 95% sensitivity and 100% specificity of the workflow to directly identify these beta-lactamases in positive blood cultures.

Of 170 positive blood cultures collected in two academic hospitals, 22 (12.9%) contained ESBL producing bacteria belonging to *E. coli or K. pneumoniae*. This percentage is higher than previously described in The Netherlands [3], with cefotaxime/ceftriaxone resistances reported to be 5% and 7% for *E. coli* and *K. pneumoniae*, respectively. However, these data were based on a larger number of laboratories, including laboratories serving non-university hospitals and general practitioners. All collected samples in our study showed full meropenem susceptibility, in agreement with the low prevalence rate of carbapenemase producing Gram-negatives in The Netherlands [3].

In the proteomic analysis of the clinical isolates, 21 out of 22 (95%) of the phenotypically ESBL-positive isolates contained a CTX-M ESBL. Cefotaximases are the most widespread ESBLs and a high representation in this study was expected [16, 17]. In our study, the CTX-M enzymes belonged to group 1 (17 out of 21; 81%) and group 9 (4 out of 21; 19%). This is in accordance with other reports [18]. Specifically, CTX-M-15 (group 1) and CTX-M-14 (group 9) are among the most prevalent enzymes [19]. Notably, in our collection there was no relation between MIC and ESBL type, especially not for ceftazidime.

Full sequence coverage is necessary to pinpoint a protein identification to a specific ESBL but in complex samples with the use of only one proteolytic enzyme, this is not feasible. Obviously, peptide fractionation or additional experiments with another proteolytic enzyme could improve the specificity of the identification. However, we opted for a simple sample preparation protocol, which is mostly constrained time-wise by the proteomic digestion step. In our approach, the sequence coverage among ESBLs in phenotypically positive isolates ranged between 38% and 88%. This coverage is in-depth enough to classify the enzymes into phylogenetic groups, such as with the cefotaximases, but single variants cannot be distinguished using this method. This is important in distinguishing beta-lactamases that have reported broad and extended-spectrum activities, based on small permutations. For example, one blood culture sample contained a SHV beta-lactamase. The sequence coverage obtained by LC-MS/MS analysis was not sufficient to distinguish between a broad and extended-spectrum beta-lactamase. The amino acid at position 238 is instrumental in cephalosporin resistance in SHV variants and the tryptic peptide covering this amino acid is therefore necessary for the unambiguous assignment of the ESBL status [20]. The corresponding tryptic peptides of the SHV-1 (broad spectrum) and SHV-2 (extended-spectrum) are TGAGER and TGASER, respectively. While the double-charged state would be within the mass range of the mass spectrometer, sensitivity in this low mass range is not optimal and short peptides can also be difficult to retain and separate in liquid chromatography. A more targeted approach might be more suitable for such specific peptides [21]. Importantly, the LC-MS/MS identified SHV beta-lactamase in isolate 15 was confirmed to be an ESBL (SHV-12) by our PCR and sequence analysis. The PCR analyses revealed three additional SHV beta-lactamases which were not identified in the proteomics analysis. Sequence analysis demonstrated that the three additional SHV beta-lactamases were non-ESBLs (SHV-1, SHV-11) and could have been missed in our proteomic analysis due to lower abundance as compared to the ESBL-SHV. Therefore, as it stands now, identifying a SHV with high expression combined with the phenotypical results indicates an ESBL-SHV, but our proteomic data was not sufficient to unambiguously draw this conclusion.

In this proof-of-principle study, ESBLs from the CTX-M group were easy to identify. PCR-based methods have been successfully applied for the identification of ESBLs in blood cultures [22, 23], and in our study, the CTX-M PCR results fully correlated with the proteomics results. The aim of our prospective study was to demonstrate the applicability in normal routine, and therefore we detected mainly CTX-M ESBL. Larger clinical sample cohorts and spiking experiments with other ESBL/carbapenemase producing bacteria in negative blood cultures are necessary to demonstrate the general applicability of our approach. Based on the results of our previous study, this workflow should also be suitable for the detection of OXA-48 and KPC beta-lactamases directly in blood cultures [11]. The sample preparation is highly similar and overall proteome and protein coverage was significantly higher using this nanoLC platform. Moreover, the mass spectrometric analysis part of our workflow can be easily exchanged for other high-end mass spectrometry analysers (such as Orbitraps) with even higher speed and sensitivities. As with all genetic methods, a positive identification does not guarantee protein expression. More sensitive proteomic analysis could therefore give some insight into our problem to detect the additional non-ESBL lactamases with our proteomics workflow.

Apart from genetic tests, there are alternative methods to detect the presence of ESBLs in blood culture bottles. Oviaño et al. monitored ESBL activity directly from blood cultures using MALDI-TOF MS by measuring antibiotic hydrolysis [24]. Reported sensitivity and specificity are high, suggesting that such an approach can be used as an alternative to traditional susceptibility testing. Moreover, hydrolysis based assays using reporter molecules are mentioned in literature and available as commercial kits [25, 26]. Even though hydrolysis based tests are useful, interpretation can be difficult in case of enzymes with a lower activity, and they provide no insight in the identity of the ESBL. In comparison with genetic and hydrolysis based methods, our workflow allows the direct identification of the enzyme responsible, providing molecular information about the phenotype.

Overall, in this proof-of-principle prospective study we demonstrate the direct identification of an ESBL in all blood cultures that contained bacteria positive for a CTX-M type ESBL. The method is specific enough to recognise specific groups of CTX-M ESBL. To improve on this proof-of-principle study in the future a number of aspects need further exploration. Among these, shortening the time-to-report and automation of the procedure are among the most critical [27, 28]. With this in mind, the developed platform can be used in the future for the direct identification of expressed beta-lactamases in blood cultures which provides detailed insight into the antibiotic resistance mechanism.
